# Incorporation of spray‐dried encapsulated bioactive peptides from coconut (*Cocos nucifera* L.) meal by‐product in bread formulation

**DOI:** 10.1002/fsn3.4120

**Published:** 2024-03-23

**Authors:** Khashayar Sarabandi, Alireaza Dashipour, Zahra Akbarbaglu, Seyed Hadi Peighambardoust, Ali Ayaseh, Hossein Samadi Kafil, Seid Mahdi Jafari, Amin Mousavi Khaneghah

**Affiliations:** ^1^ Research Institute of Food Science and Technology (RIFST) Mashhad Iran; ^2^ Department of Food Science & Technology, School of Medicine Zahedan University of Medical Sciences Zahedan Iran; ^3^ Cellular and Molecular Research Center Research Institute of Cellular and Molecular Sciences in Infectious Diseases, Zahedan University of Medical Sciences Zahedan Iran; ^4^ Department of Food Science College of Agriculture, University of Tabriz Tabriz Iran; ^5^ Drug Applied Research Center, Faculty of Medicine Tabriz University of Medical Sciences Tabriz Iran; ^6^ Department of Food Materials & Process Design Engineering Gorgan University of Agricultural Sciences and Natural Resources Gorgan Iran; ^7^ Halal Research Center of IRI, Iran Food and Drug Administration, Ministry of Health and Medical Education Tehran Iran; ^8^ Faculty of Biotechnologies (BioTech) ITMO University Saint Petersburg Russia

**Keywords:** biological stabilization, coconut peptide, covering bitterness, pan‐bread, spray‐drying

## Abstract

This study aimed to stabilize and mask the bitterness of peptides obtained from the enzymatic hydrolysis of coconut‐meal protein with maltodextrin (MD) and maltodextrin‐pectin (MD‐P) as carriers via spray‐drying. Essential (~35%), hydrophobic (~32%), antioxidant (~15%), and bitter (~45%) amino acids comprised a significant fraction of the peptide composition (with a degree of hydrolysis of 33%). The results indicated that the peptide's production efficiency, physical and functional properties, and hygroscopicity improved after spray‐drying. Morphological features of free peptides (fragile and porous structures), spray‐dried with MD (wrinkled with indented structures), and MD‐P combination (relatively spherical particles with smooth surfaces) were influenced by the process type and feed composition. Adding free and microencapsulated peptides to the bread formula (2% W/W) caused changes in moisture content (35%–43%), water activity (0.89–0.94), textural properties (1–1.6 N), specific volume (5.5–6 cm^3^/g), porosity (18%–27%), and color indices of the fortified product. MD‐P encapsulated peptides in bread fortification resulted in thermal stability and increased antioxidant activity (DPPH and ABTS^+^ radical scavenging: 4.5%–39.4% and 31.6%–46.8%, respectively). MD‐P (as a carrier) could maintain sensory characteristics and mask the bitterness of peptides in the fortified bread. The results of this research can be used to produce functional food and diet formulations.

## Graphical abstract

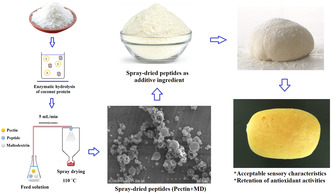

## 1 INTRODUCTION

Nowadays, the use of vegetable proteins instead of animal proteins has attracted the attention of many people, which may result from changes in the diet of some consumers to vegetarian foods, their health‐promoting effects, and lower price than animal proteins. Plant proteins can be derived from various sources, including grains and legumes, seeds of some fruits, and leaves of some plants, such as *Ramonda ser*vice (Vidović et al., 2020). On the other hand, a major challenge of the food and agriculture industries is the loss and waste of more than 30% of the produced food (annually). Most of these sources are rich in nutritious compounds (such as phenols, antioxidants, vitamins, fatty acids, minerals, fiber, protein, and carbohydrates) (Comunian et al., 2021). These sources include the waste from oil factories and their by‐products. Given the high annual production (50–60 million tons) of coconut (*Cocos nucifera* L.), the press cake obtained from the extraction of coconut milk and its oil can be considered one of the valuable sources of protein extraction (Rodsamran & Sothornvit, 2018).

In addition to nutritional importance, proteins (from various plant, animal, and marine sources) are also used as a production source of bioactive peptides. These peptides consist of several low‐molecular‐weight amino acids, which are inactive and hidden in the primary protein structure (Marciniak et al., 2018). These peptides possess multiple health‐promoting properties, such as antioxidant, antibacterial, anticancer, antihypertensive, and antidiabetic (Peighambardoust et al., 2021). Therefore, using bioactive peptides in food formulations to produce functional products can play a vital role in preventing various diseases and increasing public health (Lachowicz et al., 2021). Among food products, bread has a special place in the human diet. Therefore, its fortification can effectively provide an important part of micronutrients and health‐promoting compounds required by societies (Fitzgerald et al., 2014). Therefore, gluten‐free bread fortification with green mussel hydrolysates (Vijaykrishnaraj et al., 2016), white bread with *Palmaria palmate* (Fitzgerald et al., 2014), wheat bread with bovine globulins (Lafarga et al., 2016), and Mexican sweet bread with Lima bean or cowpea (Franco‐Miranda et al., 2017) have been investigated in various studies.

However, these compounds have practical challenges, including hygroscopicity, high bitterness, and interaction with food components (effects on the product's structure and color). These properties cause shelf life reduction and limitations in the direct use of these compounds in food products (Dayakar et al., 2022; Giroldi et al., 2022). A mechanism to reduce the mentioned disadvantages is encapsulation by the spray‐drying process, which is used to quickly stabilize various bioactive compounds by their entrapment and encapsulation in a carrier matrix (Samborska et al., 2021). To this aim, the efficiencies of various proteins, polysaccharides, and lipid carriers have been investigated for the microencapsulation of bioactive compounds. The efficiency and performance of each carrier generally depend on factors such as ease of access, price, viscosity in solution, food grading, solubility, ability to form a film/shell and increase in glass transition temperature (Tg) (Akbarbaglu, Tamjidi, et al., 2023). Moreover, the activity of carriers in preventing conformational changes and maintaining the biological activity of peptides during shear stresses and dehydration during atomization and drying is of paramount importance (Gharehbeglou et al., 2023).

The effects of different carriers on the physicochemical properties of powders obtained from protein hydrolysates have been investigated in various studies (Nurhadi et al., 2022; Qadri et al., 2022/ Choudhary et al., 2023). For example, the physicochemical stability and shelf life of microcapsules increased with spray‐drying of buffalo WPC‐hydrolysates with a blend of gum arabic‐maltodextrin carriers in a ratio of 70:30 compared to free hydrolysate; it also improved the techno‐functional parameters of the resulting microcapsule powders (Giroldi et al., 2022). In another study, spray‐dried Okara protein hydrolysate powder with maltodextrin carrier and modified starch improved the stability of the powders (at high critical RH). They increased the antioxidant capacity of the hydrolysates (Justus et al., 2022). Additionally, spray‐dried casein hydrolysate with maltodextrin and gum arabic combination carrier preserved the antioxidant activity and masked the bitter taste of bioactive peptides (Rao et al., 2016).

However, few studies are available on the effect of the encapsulation process with different carriers on preserving the properties and masking the bitterness of bioactive peptides in food products, particularly bread (Shahbazi et al., 2022; Vichakshana et al., 2022). As far as we know, the extraction of bioactive peptides from coconut pulp residues, their microencapsulation, and their application in bread formulation has yet to be studied. Therefore, the objectives of this study are: (1) protein extraction from coconut meal, enzymatic hydrolysis with alcalase, examining the composition of amino acids, and encapsulation with maltodextrin (MD) and MD‐pectin (MD‐P) carriers; (2) evaluating the effects of carriers on the physicochemical, functional, morphological, and hygroscopicity properties of peptides; (3) pan‐bread fortification with free and encapsulated peptide and investigating the microencapsulation process effect and the type of carriers on physical/textural properties, volume, porosity, and color characteristics; and (4) evaluating the antioxidant activity and sensory characteristics of fortified bread samples.

## 2 MATERIALS AND METHODS

### 2.1 Materials

The chemicals, including ABTS, DPPH, Alcalase 2.4 L., Comasi brilliant blue (G250), Apple pectin (P) with 70%–75% esterification, and Pyrocatechol violet, were sourced from Sigma–Aldrich Co (St. Louis, MO, USA). Maltodextrin (DE18‐20) was obtained from Pooran powder Co. (Isfahan, Iran). TCA (trichloroacetic acid) and other chemicals used were procured from Merck.

### 2.2 Protein extraction

The coconut oil extraction by‐product (by the cold pressing process) was purchased from a local market (Tabriz, Iran) and then ground in this study. The preparation and extraction of the protein concentrate from the coconut meal (CM) powers were performed by the method of Xu et al. (2021). Oil removal from CM powder (20% w/v mixture) was done with hexane for 4 hours. Then, the proteins in defatted powders (5% w/v dispersion in distilled water with pH = 9.5) were extracted for 1 hour. The proteins in the supernatant were precipitated with 0.5 M HCl and in the isoelectric range (pH ~ 4.5). After neutralization with 0.5 M NaOH, the precipitated proteins were freeze‐dried at −20°C under a pressure of 0.1 mbar (Christ, Germany).

### 2.3 Enzymatic hydrolysis of CM‐protein

Based on initial trial and error, 5% w/v coconut‐meal protein (CMP) solution was hydrolyzed for 2 hours at E/S = 2% v/w with Alcalase (pH = 8, 50°C). After inactivating the enzymes (95°C for 15 min), the reaction medium was centrifuged (6000 ***g*** for 10 min), and the resulting supernatant was freeze‐dried (Akbarbaglu, Ayaseh, et al., 2023).

### 2.4 Degree of hydrolysis (DH)

Suspension of CMP hydrolysate and TCA (0.44 M) were mixed at 1:1 v/v ratio and kept refrigerated at 4°C for 10 min. After centrifuging the mixture (at 7000 ***g*** for 10 min), the supernatant phase was used to evaluate the concentration of soluble peptides, before and after precipitation by TCA using the Bradford method (Bradford, 1976), using a standard curve drawn by bovine serum albumin (BSA). DH (%) was calculated by the following equation:

(1) $$\mathrm{DH}\;\left(\%\right)=\frac{\text{Protein}\;\left(\mathrm{TCA}+\text{Supernatant}\right)}{\text{Protein}\;\left(\text{hydrolysate suspension}\right)}\times 100$$

### 2.5 Amino acid composition

First, complete acid digestion of CM and hydrolyzed proteins (6 N HCl, 110°C) was performed for 24 h. The composition of amino acids was analyzed after derivatization with diethyl ethoxymethylenemalonate using an RP‐HPLC device (Novapack C18, 4 μm, Waters, Milford, MA). Tryptophan was identified and determined after alkaline hydrolysis of the samples. Then, the composition of amino acids was calculated and reported in terms of mg/g (Liu, Li, et al., 2022).

### 2.6 Spray‐drying microencapsulation

First, 30 mL of 10% (w/v) peptide solution in phosphate buffer (pH of 7.4) was mixed with an equal volume of 20% (w/v) maltodextrin solution. To investigate the impact of alternative carriers, 3 g of pectin (P) were used as a substitute for the maltodextrin. The microencapsulation process was conducted using a mini spray dryer (Büchi B‐290, Switzerland) under specific conditions, including an inlet (*T* = 140°C), an outlet (*T* = 85°C) air temperatures, a feed rate of 5 mL/min, a drying air volume of 0.56 m^3^ h^−1^, and an air pressure of 5.6 bar (Sarabandi et al., 2023).

### 2.7 Physicochemical and functional properties

Water solubility (WS), hygroscopicity, moisture content (MC), and water activity (WA) were calculated using the methods explained by Akbarbaglu, Ayaseh, et al. (2023); Akbarbaglu, Tamjidi, et al. (2023).

### 2.8 Morphological properties

The samples were coated with a thin layer of gold and then analyzed for their morphological properties using scanning electron microscopy (SEM) at an accelerating voltage of 25 kV (HITACHI PS‐230, Japan).

### 2.9 Preparation of fortified bread

In this research, the method described by Pasrija et al. (2015) was employed with certain adjustments to prepare bread dough. The formulation for 100 g of flour, consisting of 12.7% moisture, 12% protein, and 0.68% ash, involved the inclusion of 55.5 mL of water, 1.5 g of salt, 2 g of Saccharomyces cerevisiae yeast, 1 g of improving agent, and 1 g of sugar. Initially, the dry ingredients were combined using a Kenwood Ltd mixer (Hampshire, UK), following which the yeast suspension (a mixture of yeast, water, and sugar) was added to form the dough (control). To produce fortified bread, 1 g of pure peptide (sample 2), 3 g of spray‐dried peptide with MD (sample 3), and 3 g of encapsulated peptide with MD‐Pectin (sample 4) were incorporated into the other components. The selection of 3 g of encapsulated powder, with a carrier‐to‐peptide ratio of 2:1, was made to ensure the maintenance of 1 g of peptide across all formulations. Following the mixing process, an initial fermentation was conducted for 20 min at room temperature with a relative humidity of 80%. The dough was subsequently divided into 70 g portions, placed into molds, and subjected to a secondary fermentation for 50 min under similar conditions. The bread was baked in a Zuccihelli oven (Forni, Italy) at 210°C for 20 min. Finally, the bread samples were cooled at room temperature for 1 h before being packaged in polyethylene bags.

### 2.10 Fortified bread characterization

The moisture content (MC), water activity (*a*_w_) (measured on days 1 and 3), and specific bread volume were assessed following the method outlined by Schmiele et al. (2017). The porosity of the bread was determined using a digital Canon camera (Powershot A3400), an image‐capturing box, and image analyzer software (ImageJ 1.47v, USA). The texture of the bread crumbs will be evaluated using an Instron machine equipped with a 5 N load cell and a probe diameter of 36 mm. The crosshead and chart speed were set at 100 and 500 mm/min, respectively, with a 3:1 ratio of the chart to crosshead. To measure the firmness of the crumb, cubic slices (2.5 × 2.5 × 2.5 cm) were prepared and compressed to 50% of their initial height. The force (in Newtons) required for the compression was then recorded (Karimi et al., 2020).

### 2.11 Color analysis

Bread color was quantified using L*, a*, and b* values. Sample images were acquired using a Canon Powershot‐A3400 camera, and color analysis was performed using ImageJ (http://imagej.net) software, following the methodology outlined by Karimi et al. (2021). The hue angle and chroma were calculated using the following formula:

(2) $$\mathrm{Hue}=\tan^{-1}\;\left(b^{*}/a^{*}\right)$$

(3) $$\text{Chroma}={\left[{\left(a^{*}\right)}^{2}+{\left(b^{*}\right)}^{2}\right]}^{1/2}$$

### 2.12 Antioxidant characterization

First, the crumb of the breads was dried at room temperature and then ground. To evaluate the antioxidant activity, 1 g of powdered bread crumbs was extracted with 10 mL of distilled water for 30 min. The extract was centrifuged (7000 ***g***, 10 min). The supernatant solution was collected and used for antioxidant tests.

#### 2.12.1 DPPH‐radical scavenging

The mixture containing an equal volume of extract solution and DPPH (0.1 mM) was kept in the dark for 30 min. After 10 min of centrifugation at 5000 ***g***, the absorbance of the supernatant was read at 517 nm. Subsequently, the percentage of radical inhibition (RI) was computed using the following formula (Gan et al., 2020):

(4) $$\mathrm{RI}\;\left(\%\right)=\left[1-\left({\text{sample}}_{\mathrm{Abs}}/{\text{blank}}_{\mathrm{Abs}}\right)\right]\times 100$$

#### 2.12.2 ABTS radical scavenging

A mixture containing ABTS (7 mM) and potassium persulfate (2.45 mM) was prepared, kept in the darkness (12–14 h), and then diluted with 0.2 M PBS (pH 7.4 up to final absorbance of 0.70 at 734 nm). The absorbance of the extract (30 μL) reaction mixture and ABTS solution (3 mL) was read at 734 nm after 6 min of storage in the dark. Equation 4 was used to determine the percentage of ABTS radical inhibition (Liu, Wang, et al., 2022).

### 2.13 Sensory properties

Twelve semi‐trained panelists will conduct the sensory evaluation of the bread. The scoring will be based on a 5‐point hedonic scale (1 = unacceptable, 5 = very acceptable) for attributes including color, flavor, texture, chewing ability, and overall acceptance, as described by Fitzgerald et al. (2014).

### 2.14 Statistical analysis

All tests were performed in triplicate. ANOVA and comparison of means (Duncan's test) with a significance level of 5% were used to evaluate statistical differences between mean (SPSS software, version 19.0, SPSS Inc., Chicago, IL).

## 3 RESULTS AND DISCUSSION

### 3.1 Degree of hydrolysis (DH) and amino acid composition

**FIGURE 1:**
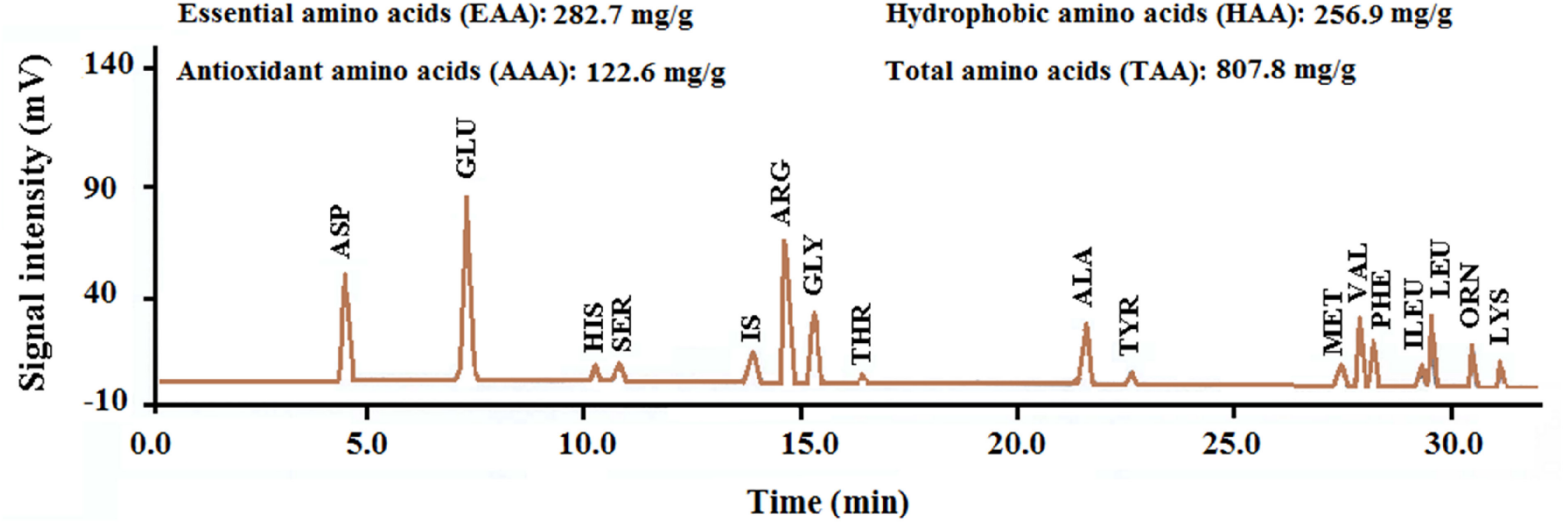
HPLC chromatogram of amino acids profile of coconut‐meal protein.

A degree of hydrolysis (DH) of about 33% was obtained for the produced peptide. The value of this index can affect the structural features, size, molecular weight, free amino acids, and biological activities of peptides (do Evangelho et al., 2017). Figure 1 shows the amino acid profile analysis in coconut protein hydrolysate. As shown in the figure, essential (~35%), hydrophobic (~32%), and antioxidant (~15%) amino acids comprise a significant part of the total composition of the peptide, with the free hydrophobic and antioxidant types comprising about 45%. On the other hand, bitter amino acids (leucine, valine, tryptophan, isoleucine, phenylalanine, and lysine) account for about 25.5% (206.2 mg/g) of the total composition of coconut protein hydrolysate (Table 1). About 42.7% of these amino acids are free, playing an effective role in the bitterness of the produced peptide (Fan et al., 2019). Therefore, these compounds' microencapsulation and bitterness control are necessary for use in food formulations due to the significant content of these amino acids in the peptide composition.

**TABLE 1:** Changes in the free amino acid (FAA) content in coconut protein (CP) after hydrolysis.

Amino acid	CP (mg AA/g dry sample)	FAA (mg AA/g dry sample)	
		0 min	120 min
Aspartic acid	82.7	0.4	10.9
Glutamic acid	161.8	2.8	17.1
Histidine	20.2	–	13.5
Serine	40.1	1.1	9.1
Arginine	112.6	0.4	26.3
Glycine	41.4	0.8	8.4
Threonine^a^	24.8	–	7.9
Alanine	38.2	–	21.2
Tyrosine	21.1	0.3	13.2
Methionine^a^	13.3	0.2	9.9
Valine^a^	36.9	–	14.3
Phenylalanine	36.2	0.7	25.1
Isoleucine^a^	24.6	1.1	6.2
Leucine^a^	47.1	2.3	23.8
Lysine^a^	32.1	1.2	15.9
Tryptophan^a^	4.7	–	2.8
HAA	222.1	4.6	116.5
AAA	91.4	1.7	55.3
TAA	737.8	11.3	225.6

^a^ Essential amino acids; Hydrophobic amino acids (HAA) = alanine, valine, isoleucine, leucine, tyrosine, phenylalanine, tryptophan, proline and methionine; Antioxidant amino acids (AAA) = tryptophan, methionine, histidine, tyrosine and lysine; Total amino acids (TAA).

The amino acid composition of hydrolysates can differ based on the type of primary protein, variety, and enzyme used for hydrolysis (Noman et al., 2018). The presence of leucine in the C‐terminal of short‐chain peptides obtained from hydrolysis is effective in antioxidant activity. Moreover, the presence of aromatic amino acids (e.g., tyrosine and phenylalanine) in coconut hydrolysate can increase antioxidant activity by transferring reactive oxygen species (ROS) (Zhang et al., 2022). Some amino acids obtained from hydrolysis have industrial applications. For example, glutamic acid is often used as monosodium glutamate (MSG) salt as a flavor enhancer in the food industry (Hajji et al., 2021).

### 3.2 Physicochemical and functional properties

The production efficiency for pure and microencapsulated peptides (with MD and MD‐P) varied by 24%–57% (Table 2). Spray‐drying without using a carrier led to the loss of many peptides. The reduction in the value of this index is influenced by factors such as adhesion and high hygroscopicity (Kumar et al., 2021). The powders' moisture content (3.4%–4.5%) and water activity (0.34–0.38) also indicated the peptides' appropriate microbial stability. The type of food composition was not significantly different in these indicators (Table 2). Solubility is an important functional indicator, particularly in producing instant powders and during restoration (Akbarbaglu, Tamjidi, et al., 2023). The value of this index was about 97% for the pure and MD‐microencapsulated peptides. However, solubility decreased to about 93% with pectin in the food composition (MD‐P). The solubility of powders depends on various factors such as the particle size of powders, the carrier type and concentration, the feed flow rate entering the chamber, temperature, pH, and ionic strength (Kumar et al., 2021). In a similar study, a solubility of >90% was reported for buffalo whey protein hydrolysates microencapsulated with MD‐GA carriers (Giroldi et al., 2022).

**TABLE 2:** Effect of carrier composition on the physical properties of spray‐dried coconut protein hydrolysate.

Carrier type	Carrier: Core	Yield (%)	Moisture (%)	Water activity	Solubility (%)	Hygroscopicity (%)
PP	0:1	24.3 ± 4.6^b^	4.5 ± 0.6^a^	0.38 ± 0.01^a^	96.9 ± 1.1^a^	39.5 ± 2.8^a^
SDP‐MD	2:1	57.1 ± 3.8^a^	3.4 ± 0.5^b^	0.34 ± 0.02^b^	97.8 ± 1.0^a^	21.3 ± 2.7^b^
SDP‐MD:P	1:1:1	54.7 ± 4.5^a^	4.2 ± 0.3^ab^	0.37 ± 0.01^a^	92.9 ± 1.3^b^	19.8 ± 2.6^b^

*Note*: Different letters in the same column indicate statistically significant differences (*p* < .05).

Abbreviations: MD, Maltodextrin; P, Pectin; PP, Pure peptide.

High hygroscopicity is a major challenge of peptides/hydrolysates. The results of hygroscopicity by peptides include phenomena such as aggregation, loss of functional properties and flowability, changes in physical nature, chemical reactions, microbial spoilage, and reduced shelf life (Sarabandi et al., 2023). The value of this index in the pure peptide (~40%) decreased significantly (to about 20%) after microencapsulation with carriers (Table 2). The use of carriers with low hygroscopicity and high Tg can play an important role in reducing the value of this index in spray‐dried peptides (Barón et al., 2021). In a similar study, the encapsulation of whey protein hydrolysates (WPH) reduced hygroscopicity by 27.4%–57.6% compared to pure hydrolysate. The value of this index increased with increasing WPH concentration in microcapsules (Rukluarh et al., 2019).

### 3.3 Morphological properties

**FIGURE 2:**
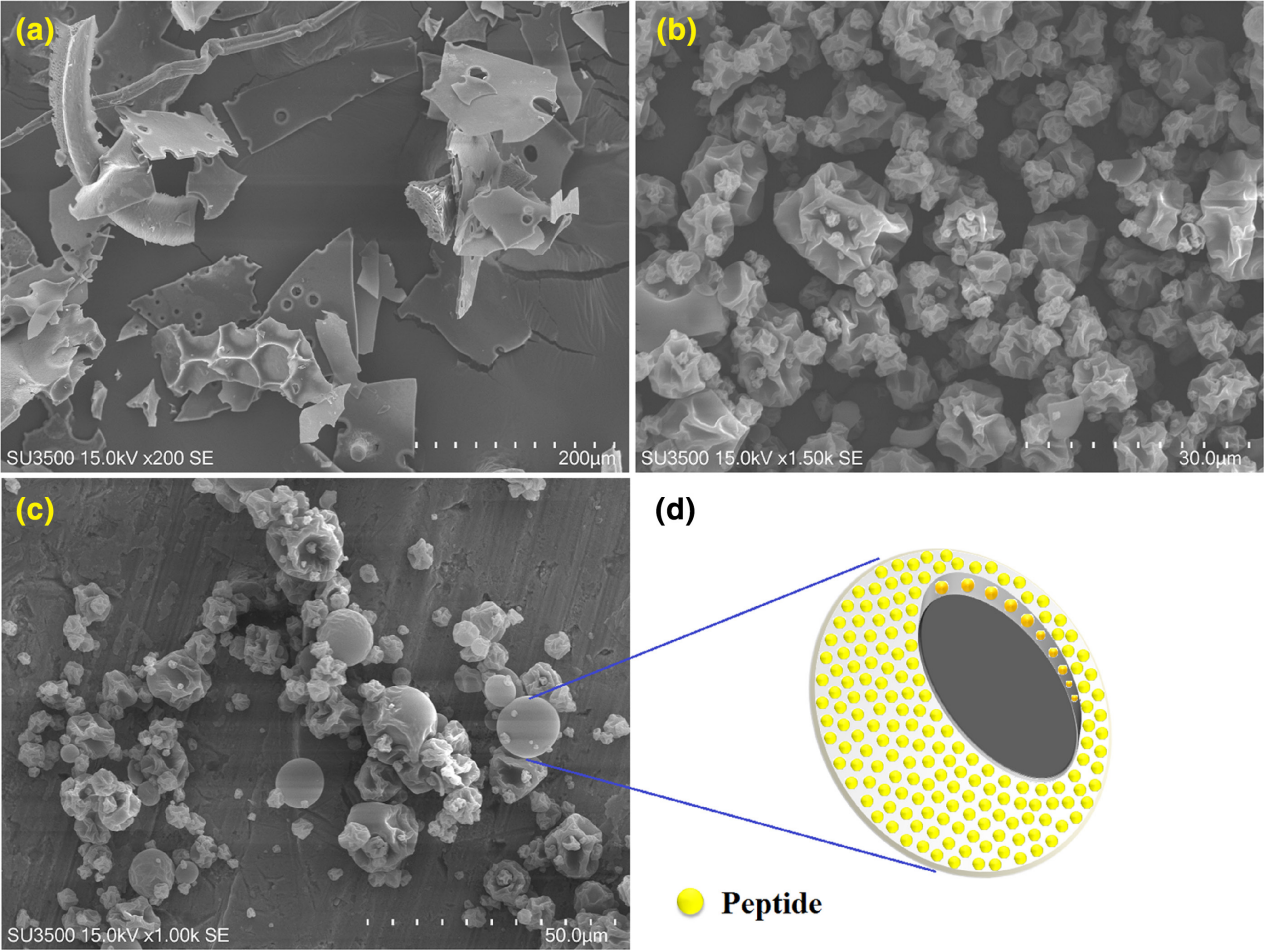
Morphological properties of (a) freeze‐dried free peptide, and spray‐dried coconut‐meal bioactive peptide with (b) Maltodextrin, (c) combination of Maltodextrin with pectin as carrier, and (d) schematic of peptides distribution within carrier matrix.

Figure 2 shows the morphological features of pure freeze‐dried peptides (Figure 2a), MD‐encapsulated samples (Figure 2b), and spray‐dried MD‐P (Figure 2c). Freeze‐dried hydrolyzed particles had porous and broken flake‐like structures with sharp edges. Flake‐like structures and broken glass in casein hydrolysate microcapsules using freeze‐drying were also reported by Rao et al. (2016). The microencapsulated peptides also had wrinkled structures with surface indentations of different sizes. The images show that the particle size distribution in MD microcapsules is more balanced than in MD‐P. Adhesion between fine and coarse particles was also observed in some areas of MD‐P‐microencapsulated samples. This result may result from colliding wet or semi‐dry particles/droplets with each other during the atomization and drying process (Giroldi et al., 2022).

Based on SEM images, hollow spherical particles indicate the distribution of produced peptides in matrix‐like structures (in the particle wall). A schematic of the distribution of peptides inside the carrier matrix is also shown in Figure 2d. Similar results were reported for *using a spray dryer for Lysiphyllum strychni folium* extract microcapsules with pectin (P: LS extract ratio 10:1 w/w) at 100°C (Goli et al., 2022). Similar structures were observed for freeze‐dried and spray‐dried *Perinereis aibuhitensis* (Liu, Wang, et al., 2022), *stripped weakfish* (Lima et al., 2019), and spent‐hen meat (Kumar et al., 2021) hydrolysates.

### 3.4 Fortified bread characterization

#### 3.4.1 Moisture contact and water activity

**FIGURE 3:**
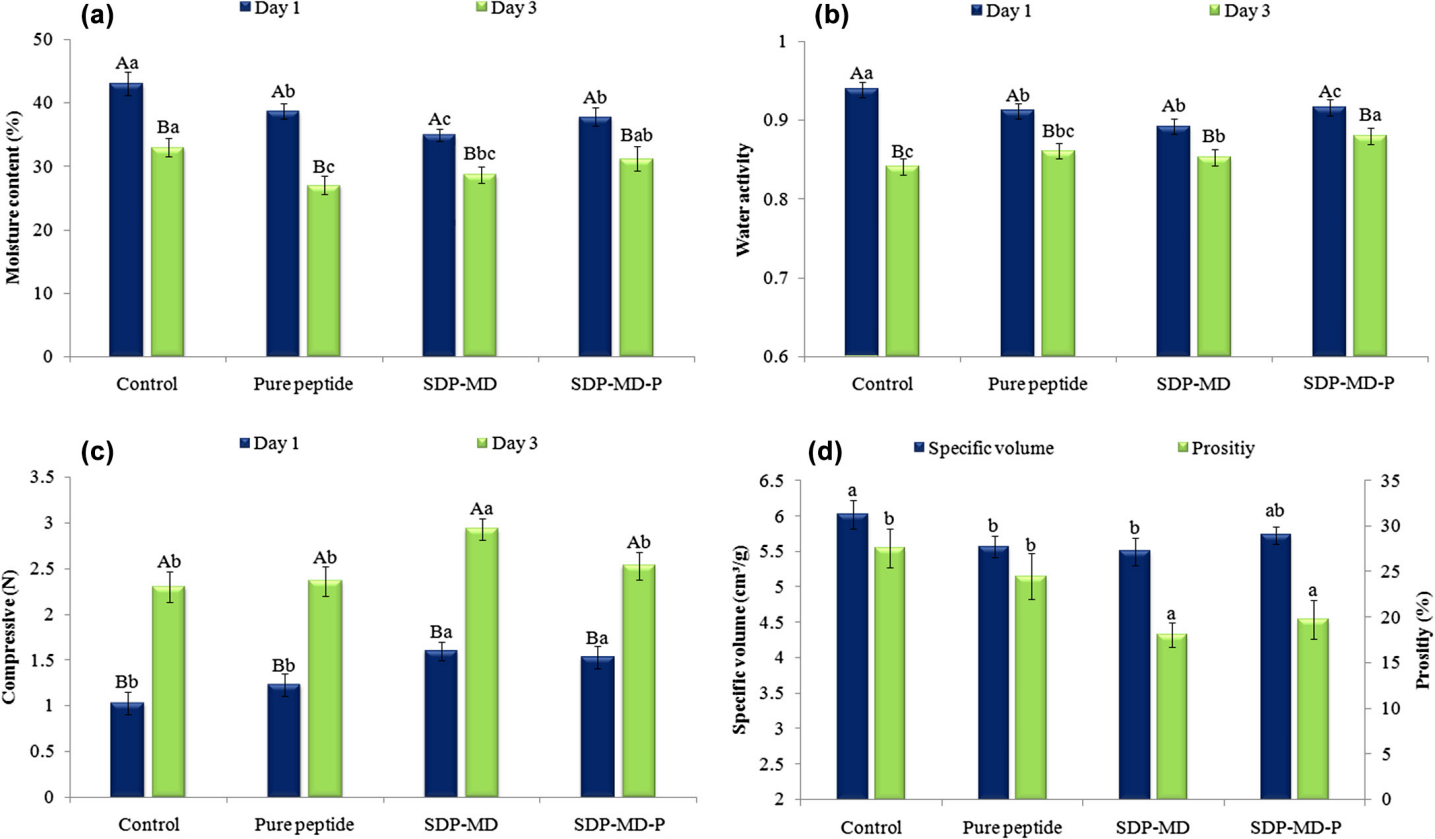
Effect of added pure and spray‐dried coconut‐meal peptide on the (a) moisture, (b) water activity, (c) texture, and (d) specific volume and porosity of fortified pan‐breads. SDP: Spray‐dried peptide with maltodextrin (MD) and Pectin (P).

The physical (moisture content and water activity) and textural (specific volume, porosity, and compression) properties of bread are shown in Figure 3. Moisture content and water activity play an important role in the shelf life of bread. According to the figure, the moisture content of control bread (43.03%) was higher than that of other bread samples (Figure 3a). The bread sample with MD‐encapsulated hydrolysate (SDP‐MD) contained the lowest moisture content (34.96%). A slight increase in the moisture content of bread samples containing pure and MD‐P‐microencapsulated hydrolysates may be caused by the high hygroscopicity of protein hydrolysates and pectin present in the microcapsule wall, respectively. Furthermore, the moisture absorption and holding capacity are mediated through the interaction of water with amine, hydroxyl, and carboxyl groups in proteins and polysaccharides (Pasrija et al., 2015). The water activity of bread samples was in the 0.89–0.93 range. Adding the hydrolysate and its microcapsules to the bread decreased the water activity of the samples compared to the control sample (Figure 3b). Besides, a decrease in the moisture content and water activity of all samples was observed after 3 days of storage at ambient temperature. This decrease can be attributed to moisture loss from the bread surface during storage and the bread staleness (Fitzgerald et al., 2014). Corresponding to these findings, a decrease in the moisture content of bread fortified with 4% WPH and 4% GPH was reported by Lafarga et al. (2016), who observed a decrease in the moisture content and water activity of all samples after storage for 6 days.

#### 3.4.2 Texture

In this study, bread hardness increased by 48% and 55% for the samples fortified with SDP‐MD‐P and SDP‐MD, respectively, compared to the control bread (Figure 3c). On the contrary, the addition of hydrolysates did not significantly affect the bread hardness compared to the control bread. The increased hardness in microcapsule‐containing bread samples can be attributed to the lack of available water for starch swelling during bread baking (Aprodu et al., 2016). El‐Sohaimy et al. (2021) attributed the elevated bread hardness to an increase in bread protein content and to a decrease in bread gluten level (responsible for bread softness) due to adding high levels of quinoa flour to the bread. Elsewhere, bread hardness decreased by adding several types of proteins along with increasing water absorption (AW) to the bread produced from rice and corn flour (gluten‐free) (Aprodu et al., 2016). The authors explained the reduced bread hardness by adding water (105%) to be associated with the denaturation of proteins and increased interaction with other bread ingredients.

In this study, bread hardness increased evidently in all bread samples during storage for 3 days. There was no significant difference in the bread hardness, except between the SDP‐MD bread sample and the control bread. The increased bread hardness may be caused by decreased moisture during storage, elevated starch retrogradation, and bread staleness. A similar result was obtained in wheat bread containing 30% buckwheat and 4% seaweed hydrolysate, and the bread hardness increased from 828 g to 2459 g during storage for 120 h (Fitzgerald et al., 2014).

#### 3.4.3 Specific volume and porosity

Bread texture is related to its moisture content and mechanical properties (Karimi et al., 2021). The specific bread volume decreased by adding pure hydrolysate powder and microcapsules compared to the control bread (Figure 3d). Encapsulation and the carrier type influenced the bread‐specific volume compared to the sample containing pure hydrolysate. The decreased bread volume can be associated with the participation of hydrolysates in the gluten network structure. These compounds may break the gluten network integrity and reduce its ability to preserve fermentation gases (El‐Sohaimy et al., 2021). Likewise, Franco‐Miranda et al. (2017) reported that adding 1% and 3% cowpea hydrolysate reduced the bread‐specific volume to 34.7 and 29.6 mL/g, respectively, compared to the control bread (49.7 mL/g). Bread fortification with quinoa flour at high proportions produced a fibrous gluten network. It reduced the specific volume of the bread, which was attributed to the presence of dietary fiber in quinoa flour and the gluten network dilution due to the deficiency of gluten components (glutenin and gliadin) (Xu et al., 2019). However, El‐Sohaimy et al. (2021) did not observe a difference in bread‐specific volume by adding 5%–30% quinoa flour.

Porosity in bread is caused by CO_2_ gas production by yeasts and, to a small extent, by the hetero‐fermentative activities of lactic acid bacteria (LAB) (García‐Segovia et al., 2017). The presence of many bubbles with smaller sizes and thinner walls can produce bread with an elastic and softer texture (Xu et al., 2019). As shown in Figure 3d, adding hydrolysates to the bread did not produce a difference in porosity compared to the control bread. Nevertheless, the porosity decreased from 27.6% (control bread) to 18.1% and 19.8% for SDP‐MD and SDP‐MD‐P bread samples, respectively. Adding dairy ingredients (milk or acid whey) at ratios of 12.5%–50% to the bread led to a decrease in porosity from 77.23% (in control bread) to about 70.3%–75.7% in other bread samples (Iuga et al., 2020). They attributed this decrease to an increase in viscosity due to the gelling ability of milk proteins. In another study, porosity was affected by the buckwheat flour percentage added to wheat flour and the flour particle size of buckwheat. The porosity was reduced in the resulting bread by adding high proportions of buckwheat flour (10%–20%) with a smaller particle size (<180 μm) (Coţovanu & Mironeasa, 2022).

#### 3.4.4 Color analysis

The color of the product greatly influenced its consumer acceptance. The color of the bread samples changed slightly by adding the hydrolysate and its microcapsules (Table 3). A decrease in lightness (L index) was observed by adding pure hydrolysate and microcapsules to the bread. The bread containing pure hydrolysate recorded the lowest L index (62.5). According to a report (Mariscal‐Moreno et al., 2021), products with an L index in the 50–100 range are considered bright products. Positive values of *b* and indices showed decreased yellow and increased red, respectively, by adding hydrolysate powder and its microcapsule compared to the control sample. The yellow color of the control bread sample is associated with the wheat flour yellowness used in the bread. The bread color, particularly the bread crust, depends on the dough properties, such as the moisture content, the amino acid content, reducing sugars, and baking operating conditions (such as baking temperature, relative humidity of the oven air, and heat transfer rate) (García‐Segovia et al., 2017). The darkened bread color may result from an increase in the bread protein content because of adding hydrolysates and the microcapsules (El‐Sohaimy et al., 2021) and/or as a result of the Maillard reaction (Marti et al., 2018). Among the samples, the color of the sample containing microencapsulated powder (MD) was less different than the control sample, probably resulting from the effect of the maltodextrin bright color in the wall of the microcapsules on the bright color of the resulting bread. Furthermore, the measured Hue and Chroma indices revealed the lowest Hue angle (tendency to red color) and the highest Chroma (color intensity) in the bread sample with pure hydrolysate among all the samples. The tendency to yellow color was evident in the control bread sample with the highest Hue angle (88.68°).

**TABLE 3:** Color properties of control and bread fortified with spray‐dried coconut protein hydrolysate.

Treatments	L*	a*	b*	Hue angle (°)	Chroma
Control	77.83 ± 1.25^a^	1.33 ± 0.02^d^	59.79 ± 1.23^a^	88.68 ± 0.07^a^	59.81 ± 1.22^ab^
F‐PP	62.51 ± 2.03^c^	22.34 ± 1.66^c^	56.78 ± 1.41^ab^	68.55 ± 0.97^d^	61.04 ± 1.91^a^
F‐SDP‐MD	73.59 ± 2.31^b^	3.56 ± 0.15^a^	57.05 ± 1.94^ab^	86.39 ± 0.08^b^	57.17 ± 1.96^bc^
F‐SDP‐MD‐P	70.97 ± 1.67^b^	5.62 ± 0.16^b^	55.36 ± 2.41^b^	84.16 ± 0.24^c^	55.64 ± 2.43^c^

*Note*: Different letters in the same column indicate statistical significant differences (*p* < .05).

Abbreviations: F‐SDP, Fortified with spray‐dried peptide; MD, Maltodextrin; P, Pectin.

#### 3.4.5 Antioxidant characterization

**FIGURE 4:**
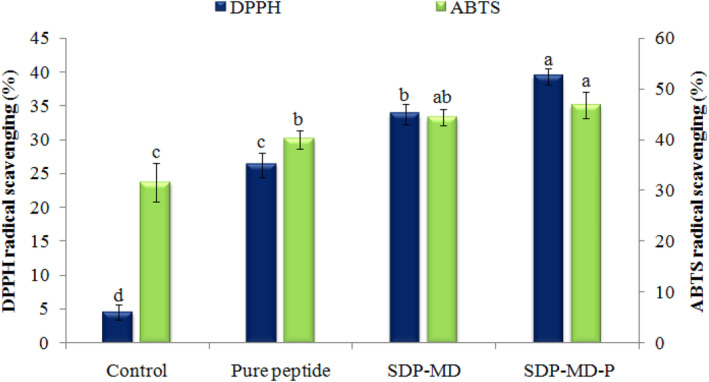
Effect of added pure and spray‐dried coconut‐meal peptide on the DPPH and ABTS radical scavenging activity of fortified pan‐breads. SDP: Spray‐dried peptide with maltodextrin (MD) and Pectin (P).

The antioxidant activity of control and fortified bread samples with the pure peptide (PP) and spray‐dried peptide (SDP) was investigated using two DPPH and ABTS radical scavenging indicators (Figure 4). Elevated DPPH and ABTS radical scavenging (5.8–8.7 and 1.27–1.48 times, respectively) was observed in fortified bread samples compared to the control bread, which may be related to the antioxidant activity of enzymatic hydrolysis‐derived peptides. Similarly, hydrophobic and free aromatic amino acids in coconut hydrolysates can inhibit free radicals because of their ability to donate electrons and protons (Akbarbaglu et al., 2022). The use of hydrolyzed microcapsules in bread increased DPPH and ABTS radical scavenging (1.28 and 1.11 times, respectively) in SDP‐MD bread and in SDP‐MD‐P bread (1.50 and 1.17 times, respectively) compared to pure hydrolysate‐containing bread. The higher antioxidant activity of encapsulate‐containing bread samples suggests better preserving bioactive peptides (thermal stability) during the bread‐baking process. The utmost DPPH and ABTS radical scavenging belonged to the SDP‐MD‐P bread sample, indicating the role of using combined MD and Pectin as a wall composition in the better preservation of antioxidant compounds (Souza et al., 2017). Based on a previous report (Xu et al., 2019), bread samples containing quinoa flour showed about 1.38 and 1.45 times increase in DPPH and ABTS radical scavenging, respectively, compared to the control bread (without quinoa flour addition). In addition to the bioactive peptides' antioxidant activity, the bread samples' increased antioxidant activity can be affected by the production of some antioxidant compounds resulting from the Maillard reaction during bread baking (Lachowicz et al., 2020).

#### 3.4.6 Sensory properties

**FIGURE 5:**
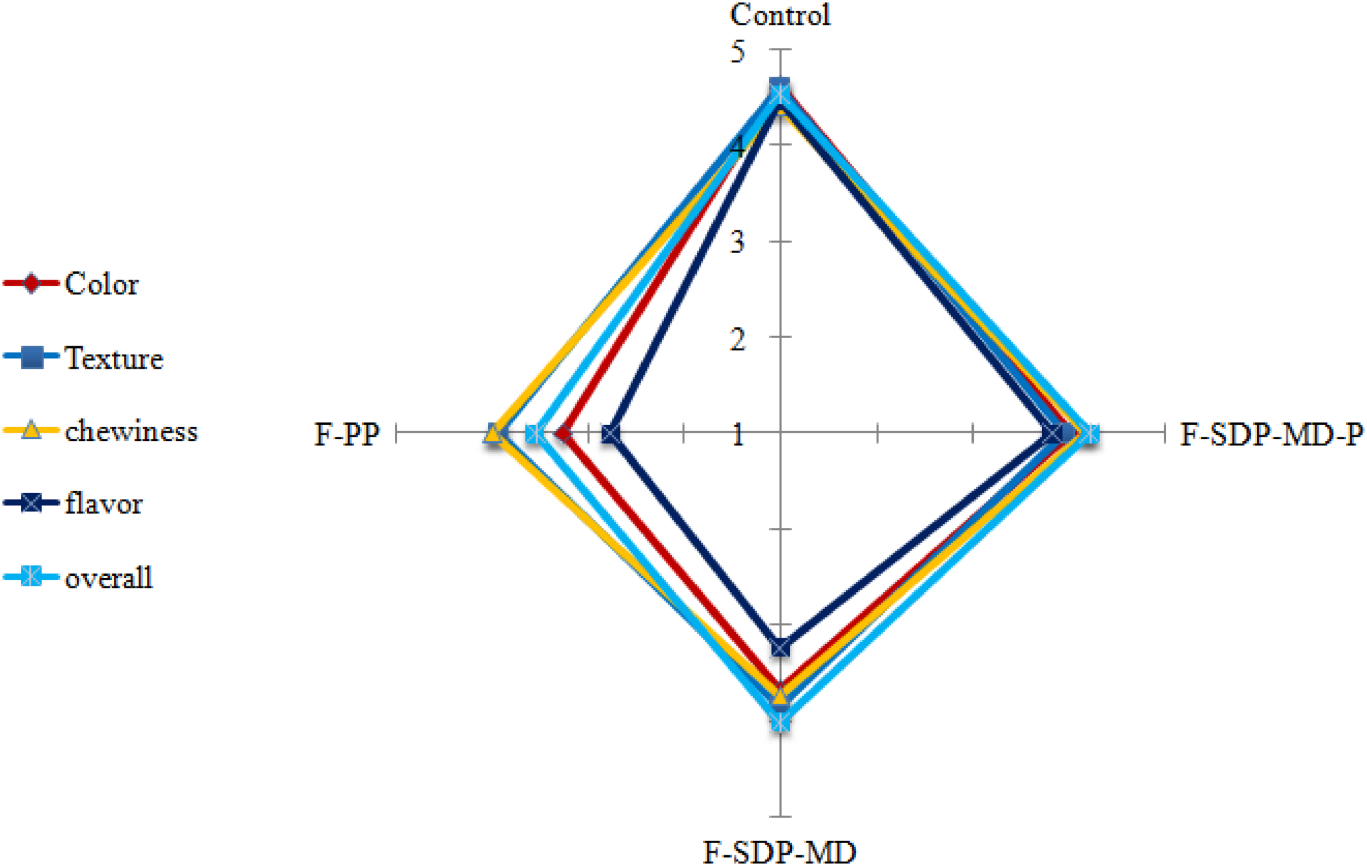
Sensory properties of control and pan‐breads fortified with free and spray‐dried coconut‐meal peptide.

Figure 5 compares the sensory characteristics (color, texture, chewiness, flavor, and overall acceptance) of the bread samples with the control bread. The color of the SDP‐MD‐P‐containing bread gained the highest score (4.03) after the control bread (4.63). Adding the hydrolysate and microcapsules to the bread increased the bread firmness, but the control bread texture was less different from that of the SDP‐MD‐P and PP bread samples. Control and fortified (SDP‐MD‐P) bread samples gained higher scores for chewiness, and the lowest score (3.73) belonged to the SDP‐MD‐containing bread. The points given to the bread flavor indicated that encapsulation significantly affected flavor acceptance. SDP‐MD‐P bread received the highest score after the control sample. According to the sensory results, SDP‐MD‐P bread had a better flavor (for masking the bitterness of peptides) than the other two bread samples (SDP‐MD and PP). Based on a report by Fávaro‐Trindade et al. (2010), flavor improvement in SDP‐MD‐P bread can be associated with the effect of encapsulation on masking the bitter taste (caused by the presence of some hydrophobic amino acids in hydrolysates) (Vijaykrishnaraj et al., 2016). The addition of 5% quinoa flour generally produced no difference in the bread sensory characteristics compared to the control bread, but adding higher percentages reduced the appearance, taste, and texture scores of the produced bread compared to the control bread (Xu et al., 2019). In our study, the highest overall acceptance belonged to the SDP‐MD‐P bread after the control bread. Vijaykrishnaraj et al. (2016) observed an increase in bread texture hardness by adding green mussel protein hydrolysates to the bread, especially at a 10% hydrolysate ratio (GMPH); however, the overall quality of the bread was accepted by the panelists.

## 4 CONCLUSION

Currently, the extraction and evaluation of bioactive peptides from different plant, animal, and marine sources are being investigated in many studies. Despite the numerous health‐promoting benefits, storing and using these peptides in food formulations is challenging because of their physicochemical instability and high bitterness. This study utilized the coconut oil extraction residue (as a by‐product and waste) for enzymatic hydrolysis and peptide production. Examination of the amino acid composition indicated the appropriate nutritional quality of the produced peptides. MD‐P carriers were used by the spray‐drying method to stabilize and mask the bitterness of peptides. The results demonstrated an improvement in production yield and physicochemical, functional, and stability properties of peptides after encapsulation. Moreover, the MD‐P combination maintained antioxidant activity (thermal stability) and the bitterness masking of peptides in the fortified bread. The results of this research can be used to fortify food formulations and produce functional products. However, more research is required to evaluate these peptides' health‐promoting properties and stability in digestive conditions, as well as using other carriers (as stabilizers).

## AUTHOR CONTRIBUTIONS

**Khashayar Sarabandi:** Data curation (equal); formal analysis (equal); funding acquisition (equal); investigation (equal); project administration (equal); supervision (equal); validation (equal); visualization (equal); writing – original draft (equal); writing – review and editing (equal). **Alireaza Dashipour:** Data curation (equal); formal analysis (equal); funding acquisition (equal); investigation (equal); software (equal); supervision (equal); validation (equal); visualization (equal); writing – original draft (equal). **Zahra Akbarbaglu:** Conceptualization (equal); data curation (equal); formal analysis (equal); funding acquisition (equal); resources (equal); validation (equal); visualization (equal); writing – original draft (equal). **seyed hadi peighambardoust:** Formal analysis (equal); funding acquisition (equal); investigation (equal); methodology (equal); project administration (equal); supervision (equal); visualization (equal); writing – original draft (equal); writing – review and editing (equal). **Ali Ayaseh:** Conceptualization (equal); resources (equal); software (equal); supervision (equal); validation (equal); visualization (equal); writing – original draft (equal); writing – review and editing (equal). **Hossein Samadi Kafil:** Funding acquisition (equal); investigation (equal); methodology (equal); project administration (equal); validation (equal); visualization (equal); writing – original draft (equal). **Seid Mahdi Jafari:** Formal analysis (equal); funding acquisition (equal); investigation (equal); project administration (equal); resources (equal); supervision (equal); validation (equal); visualization (equal); writing – review and editing (equal). **Amin Mousavi Khaneghah:** Investigation (equal); project administration (equal); supervision (equal); validation (equal); writing – review and editing (equal).

## CONFLICT OF INTEREST STATEMENT

None.

## Data Availability

The data that support the findings of this study are available upon reasonable request from the corresponding author.
